# Editorial: Experimental models of epilepsy and related comorbidities, Volume II

**DOI:** 10.3389/fphar.2023.1182958

**Published:** 2023-03-24

**Authors:** Mohd Farooq Shaikh, Irma Wati Ngadimon, Ayanabha Chakraborti, Jafri Malin Abdullah, Emilio Russo

**Affiliations:** ^1^ School of Dentistry and Medical Sciences, Charles Sturt University, Orange, NSW, Australia; ^2^ Neuropharmacology Research Laboratory, Jeffrey Cheah School of Medicine and Health Sciences, Monash University Malaysia, Bandar Sunway, Malaysia; ^3^ Department of Translational Neurosciences, College of Medicine Phoenix, University of Arizona, Phoenix, AZ, United States; ^4^ Brain Behaviour Cluster and Department of Neurosciences, School of Medical Sciences/Hospital Universiti Sains Malaysia, Universiti Sains Malaysia, Kubang Kerian, Kota Bharu, Malaysia; ^5^ Magna Græcia University, Catanzaro, Italy

**Keywords:** epilepsy, comorbidities, experimental, clinical, translational

This Research Topic is comprised of six articles, including two original research, one systematic review and three mini-review, contributions from prominent scientists in the field. The Research Topic provides an up-to-date understanding of the latest approaches in experimental and clinical epilepsy studies as well as related comorbidities. The summary of each of these articles is encapsulated below.

Extensive evidence describes teratogenicity in pregnant women with epilepsy with the use of anti-seizure medications (ASM). Castro et al. examine the possible association between ASM, ion channels, synaptic proteins and embryonic teratogen. ASMs act on ion channels and proteins involved in relevant signalling and cellular responses during embryonic development, which could explain foetal malformations observed with their use. This article reviews the expression and function of these channels and neurotransmitters during embryonic development and how perturbations in their activity by ASMs may lead to neural tube defects and other major congenital malformations. Understanding how ASMs affect foetal development could improve treatment strategies for pregnant women with epilepsy.

Epilepsy is commonly associated with brain tumours due to various biological and molecular factors. Sánchez-Villalobos et al. provide an overview of ASM used to treat brain tumour-related epilepsy (BTRE) and highlights the strengths and weaknesses of different ASMs such as levetiracetam, brivaracetam, valproic acid, and sodium channel blockers like lacosamide, dibenzazepines, and lamotrigine. The choice of medication should be made based on the specific features of each patient. The study also addresses pertinent questions, such as identifying the best candidates for ASM prescription and determining the optimal time to initiate ASM treatment.

The study by Kandeda et al. investigates the potential of *P. biglobosa*, a leguminous tree commonly found in West Africa and the northern part of Cameroon, for treating drug-resistant Temporal Lobe Epilepsy (TLE). The plant contains secondary metabolites that have antioxidant and anti-inflammatory properties. The extract protected mice against stages 3 and 4 of seizures during the PTZ-kindling period and alleviated working memory impairment and anxiety-like behaviour. These findings suggest that *Parkia biglobosa* has the potential as a natural source of anticonvulsant, cognitive-enhancing agents and anxiolytic-like effects.

A network meta-analysis by Huo et al. aimed to determine the optimal ASM for preventing posttraumatic epilepsy (PTE) in patients with traumatic brain injury (TBI). A total of 25 trials were analysed, including seven randomised controlled trials (RCT) and 18 non-RCT. Results showed that both levetiracetam (LEV) and phenytoin (PHT) effectively prevented early and late PTE, with PHT also reducing mortality rates. However, both LEV and PHT had higher treatment-related adverse effects than placebo, and LEV had a slightly lower incidence of adverse effects than PHT. Based on these findings, the authors recommend LEV as the best treatment option for TBI patients but note the need for further high-quality RCT to confirm these findings.

The genus Artemisia is a widely used plant group in traditional medicine reported to have antiepileptic effects. Sailike et al. summarise 18 pharmacological studies on the antiepileptic properties attributed to its bioactive components. The genus extracts possess antioxidant, anti-inflammatory, neurotransmitter-modulating, anti-apoptotic, anticonvulsant, and pro-cognitive properties by modulating oxidative stress, protecting mitochondrial membrane potential, upregulating GABA-A and nACh receptor activities, and interfering with various signalling pathways. The authors suggest further investigations on the purification and identification of the most biologically effective compounds of Artemisia to cure epilepsy and other neurological diseases.

A retrospective study by Yu et al. elucidates the therapeutic effect of perampanel (PER) on electrical status epilepticus during sleep (ESES) in paediatric patients with focal epilepsy. Results showed that the response rate was 53.7%, with ESES resolved in 29 children after 6 months of PER add-on treatment. The study concludes that PER add-on treatment can be used as an alternative to corticosteroids and benzodiazepines for ESES. However, a longer ESES duration before a PER administration is a risk factor for PER treatment failure.

The editorial team thank all the contributing authors, associate editors and review editors for their scholarly contributions. We are hopeful that these papers would promote and inspire other researchers in this exciting research field. These articles will also contribute to the understanding of the disease progression and potentially lead to development of novel, safe and effective therapeutics to address epilepsy and associated comorbidities.

